# Spectrum of High Resolution Computed tomography chest findings in PCR positive COVID-19 patients according to duration of infection and CT severity score assessment

**DOI:** 10.12669/pjms.38.1.4204

**Published:** 2022

**Authors:** Nadia Irshad, Nadia Hanif

**Affiliations:** 1 Dr. Nadia Irshad, FCPS. Senior Registrar, Department of Radiology, PGMI/AMC/Lahore General Hospital, Lahore, Pakistan; 2 Dr. Nadia Hanif, FCPS. Assistant Professor, Department of Radiology, PGMI/AMC/Lahore General Hospital, Lahore, Pakistan

**Keywords:** COVID-19, High Resolution Computed tomography (HRCT), CT Severity Score (CT-SS), Ground Glass Opacity (GGO)

## Abstract

**Objective:**

To evaluate the spectrum of HRCT findings of COVID-19 in RT-PCR positive patients according to duration of infection and severity of disease.

**Methods:**

This retrospective study was conducted at Radiology department of Lahore General Hospital, Lahore from May to July 2020. Total 40 COVID-19 patients were reviewed for clinical features, HRCT chest findings based on time from symptom onset and CT conduction. Chi-square and fissure exact test were used for measuring association with severity of COVID-19, p value ≤0.05 was reported significant. Mean CT scores were calculated. ROC curve analysis showed threshold values of CT-SS for severe disease.

**Results:**

Of total 40 patients with age ranged from 22-83 years, 22(55%) were males and 18(45%) females. The hallmark of COVID-19 was combined GGO and consolidation, GGO alone and consolidation alone in bilateral, sub pleural and posterior distribution. Early stage had normal CT or GGO alone, intermediate and late stage had both GGO and consolidation. Septal lines/bands and crazy paving pattern were prevalent in late stage. Clinically, 24 (60%) were in severe group and 16(40%) in mild group. Severity of COVID-19 was associated with GGO alone (p=0.05), GGO and consolidation (p=0.01), crazy paving (p=0.01) and lung scores (p≤0.05). The threshold values of CT-SS for identifying severe disease by two radiologists were 18.50 and 20.50.

**Conclusion:**

HRCT manifestations along with CT-SS aids in predicting disease severity. Staging according to duration of infection is effective in understanding variation in pattern of chest findings in coronavirus disease.

## INTRODUCTION

The city of Wuhan in China became the epicenter of spread of novel COVID-19 infection. Progressing from an epidemic to a global pandemic, this ailment penalized the mankind within no time. Due to rapid transmission via air-borne droplets and highly virulent nature of virus, a large number of patients have emerged creating a havoc. SARS- CoV-2 was declared new name for causative agent of coronavirus disease due to genetic relation with SARS.^1^

Apart from the reported high sensitivity of CT scan as compared to RT-PCR, CT has proved to be an ideal tool for monitoring the disease course.^2^ COVID-19 presents with fever of varying degrees, cough, dyspnea, diarrhea, change of sense of taste or smell, and myalgia. Many asymptomatic carriers are also identified. Elderly with co-morbids is at a greater risk of acquiring severe infection with resultant mortality.^3^ Different CT severity scoring systems are identified based on lobes and segments involved which in conjunction with clinical classification evaluates disease severity and guides in better treatment strategy.^4^,^5^ Timing of CT conduction from onset of symptoms is vital due to diverse radiological appearances at different stage of disease.^6^

CT chest findings of bilateral, multi-lobar GGO and/or multi-focal consolidations in sub-pleural location is considered pathognomic for COVID -19. With progression of disease septal thickening prevails on background of GGO manifesting as crazy paving appearance. Majority of patients recovers leaving sub-pleural bands and fibrosis, while critically ill ends in white-out lung.^7^ Meng et al. and Hu et al reported GGO alone in 94.8% and 50% asymptomatic patients respectively.^8^,^9^ Wang described temporal changes of coronavirus disease in serial CT scans enabling better staging.^10^

Our study targeted to evaluate the spectrum of HRCT findings of COVID-19 according to duration of infection. The detailed CT-SS assessment was aimed to predict clinical severity so that strategy is formulated regarding its management to decrease the morbidity and mortality.

## METHODS

After approval from Ethical Review Board of Lahore General Hospital (00-171-20 dated:15-9-2020), this retrospective study was conducted from May to July, 2020 in 40 patients aged 22 to 83 years who were tested positive for COVID-19 by RT-PCR along with simultaneous conduction of HRCT chest in Radiology Department. These patients presented in either outpatient or emergency with symptoms of fever, cough/sore throat, or shortness of breath. PCR negative patients with positive HRCT chest findings for COVID-19 were excluded.

All images were taken on 128 slice CT scanner with patient in supine position and scanning done from lung apices to costo-phrenic angles. The main scanning parameters were 120 KVP, 450 mAs, pitch 1.4, FOV of 406 mm and slice thickness of 1mm. CT was done without contrast. Images were sent to workstation and picture archiving and communication systems (PACS).

Two radiologists with seven and nine years of experience respectively reviewed all HRCT Chest images and described the findings as unilateral or bilateral ; pattern of involvement as GGO alone, combined GGO with consolidation, consolidation alone, crazy paving pattern, vascular enlargement, round opacity, reverse halo, sub-pleural lines/bands; segments and lobes involved; distribution as sub pleural/ peripheral, anterior or posterior, perihilar/ central, bronchovascular or diffuse.^11^ Associated findings as emphysema, bronchiectasis, pleural effusion or lymphadenopathy were noted. Time between onset of symptoms and CT conduction was determined and patients were divided into early (0-2 days), progressive (3-5 days) or late (6-12 days) stage of disease.^12^

According to WHO clinical classification, patients were divided into mild, moderate, severe or critical stages. Mild patients had clinical symptoms without evidence of viral pneumonia or hypoxia. Moderate disease had clinical signs of pneumonia with respiratory rate <30 breaths / minutes and oxygen saturation >90%. Severe cases have clinical signs of pneumonia with respiratory rate >30 breaths / min and oxygen saturation <90%. Critical cases have septic shock, acute respiratory distress syndrome or needs mechanical ventilation.^13^,^14^ For sake of convenience, mild and moderate groups were merged while severe and critical cases were also combined into single group in this study. The CT-SS describes extent of involvement of 20 lung segments with 0: no parenchymal involvement, 1: <50% parenchymal involvement and 2: >50% parenchymal involvement. Total score is obtained by adding individual segments score ranging from 0 to 40.^15^

The collected data was entered into SPSS 23 version and analyzed. Quantitative variable including age and CT-SS were described as mean or standard deviation. Qualitative variables including clinical history, HRCT chest findings, lung lobes and segment involvement were described as frequency and percentages. CT-SS in mild and severe group were compared. ROC curve was drawn with threshold value for severe disease determined along with calculation of area under the curve. The inter- rater reliability score between two radiologists was calculated.

## RESULTS

Out of total 40 patients, 22(55%) were males and 18(45%) were females. Age ranged from 22 to 83 years with mean age of 50 years±14 years. Fever was seen in 39(97.5%), cough in 27(67.5%) and shortness of breath in 24(60%) patients. Diabetes and hypertension were the major comorbidities 5(12.5%) (Table-I).

**Table I:** Demographic and Clinical features of COVID-19.

Age groups	Frequency
20-40y	11(27.5%)
41-60y	22(55%)
61-80y	6(15%)
>80y	1(2.5%)
***Symptoms***	
Fever	39(97.5%)
Cough	27(67.5%)
Sputum	1(2.5%)
Loss of taste or smell	8 (20%)
Shortness of breath	24(60%)
***Respiratory rate***	
<30	17(42.5%)
>30	23(57.5%)
***Oxygen Saturation***	
>93%	18(45%)
<93%	22(55%)
***Comorbidities***	
Diabetes & Hypertension	5(12.5%)
Diabetes	4(10%)
Diabetes & Ischemic heart disease	3(7.5%)
Hypertension	3(7.5%)
Pancreatitis	1(2.5%)
Liver parenchymal disease	1(2.5%)

The most frequent finding on HRCT chest was combined GGO and consolidation 19(47.5%) with posterior and sub-pleural distribution 37(92.5%). Bilaterality was seen in 37(92.5%) while one (2.5%) had unilateral finding. Associated findings were lymphadenopathy 11(27.5%), pleural effusion 6(15.0%), bronchiectasis 5(12.5%) and emphysema 2(5.0%).

**Fig.1:**
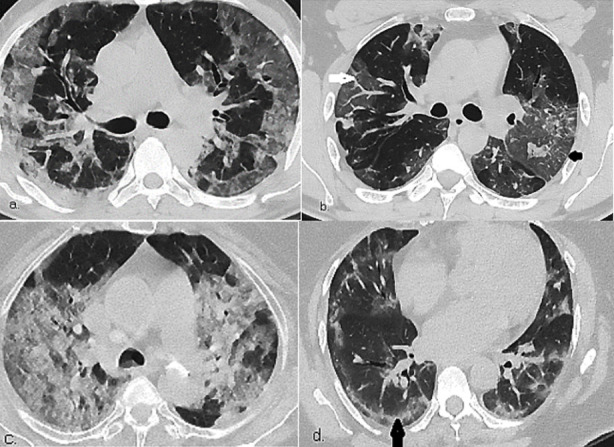
Axial HRCT Chest images of four different patients a) bilateral sub-pleural GGO and consolidation in posterior distribution b) Focal septal thickening within GGO (Black arrow). Vascular enlargement also seen (white arrow) c) Diffuse crazy-paving pattern and consolidation d) Sub-pleural line/band in peripheral distribution at late stage of disease.

Among two patients of early stage (0-2 days), one had normal HRCT, while other had GGO alone. Consolidation, crazy-paving and vascular enlargement were absent. Of nine patients of progressive stage (3-5 days), combined GGO and consolidation 4(44.4%) and GGO alone 3(33.3%) were seen. During late stage (6-12 days), among 29 patients, combined GGO and consolidation 15(51.7%), sub-pleural lines/bands 14(48.3%), vascular enlargement 13(44.8%) and crazy paving pattern 11(37.9%) were noted. The distribution of disease was posterior, sub-pleural in 29(100%), bronchovascular in 20(69.0%), peri-hilar in 13(44.8%) and diffuse in 6(20.7%) during late stage. (Table-II & Fig.1). Severity of COVID-19 was associated with GGO alone (p=0.05), GGO and consolidation (p=0.01), crazy paving (p=0.001), vascular enlargement (p=0.005) and lung segment scores (p≤0.05).

**Table II:** HRCT Chest findings of coronavirus disease according to duration of infection.

CT Findings		Total N=40	Early (0-2 days) n=2	Progressive (3-5 days) n=9	Late (6-12days) n=29
GGO alone		15 (37.5%)	1(50%)	3(33.3%)	11(37.9%)
Consolidation alone		6(15%)	0	1(11.1%)	5(17.2%)
GGO & consolidation		19(47.5%)	0	4(44.4%)	15(51.7%)
Pattern of consolidation	Air bronchogram	13(32.5%)	0	3(33.3%)	10(34.5%)
	Segmental	4(10%)	0	2(22.2%)	2(6.9%)
	Sub segmental	17(25%)	0	2(22.2%)	15(51.7%)
	Segmental & sub segmental	2(5%)	0	0	2(6.9%)
Crazy paving pattern		12(30%)	0	1(11%)	11(37.9%)
Sub-pleural lines/bands		14(35%)	0	0	14(48.3%)
Vascular enlargement		15(37.5%)	0	2(22.2%)	13(44.8%)
Round opacity		5(12.5%)	0	3(33.3%)	2(6.9%)
Reverse Halo		1(12.5%)	0	1(11.1%)	0
Distribution	Posterior	37(92.5%)	1(50%)	7(77.8%)	29(100%)
	Anterior	27(67.5%)	0	3(33.3%)	24(82.8%)
	Sub-pleural	37(92.5%)	1(50%)	7(77.8%)	29(100%)
	Central /perihilar	15(37.5%)	0	2(22.2%)	13(44.8%)
	Broncho-vascular	24(60%)	0	4(44.4%)	20(69%)
	Diffuse	8(20%)	0	2(22.2%)	6(20.7%)

Clinically, 24 patients (60%) were in severe group and 16(40%) in mild group. Frequency of lobe involvement was right lower lobe 37(92.5%), left lower lobe 35(87.5%), right upper lobe 32(80%), right middle lobe in 32(80%) and left upper lobe 31(77.5%). On evaluation of CT-SS, most frequently involved segment was lateral segment of left lower lobe 34/40(85%), posterior and medial segments of right lower lobe 33/40 (82.5%), posterior segment of left lower lobe 33/40 (82.5%). Total mean CT-SS was 20±11.5. Mean CT score of mild groups was 10±7.4 and severe group was 27±7.6 (Table-III).

**Table III:** CT-SS in mild & severe groups in different lung segments.

Right Lung			Severity of COVID				Left Lung		Severity of COVID			

			Mild/ Moderate	Severe	Total	P-Value			Mild/ Moderate	Severe	Total	P-Value
UPPER LOBE	Apical Segment	0	16(40%)	5(12.5%)	21(52.5%)	0.01	UPPER LOBE	Apical Segment	0	18(45%)	4(10%)	22(55%)	0.000
		1	4(10%)	9(22.5%)	13(32.5%)				1	2(5%)	14(35%)	16(40%)	
		2	0	6(15%)	6(15%)				2	0	2(5%)	2(5%)	
	Anterior Segment	0	13(32.5%)	1(2.5%)	14(35%)	0.000		Anterior Segment	0	14(35%)	1(2.5%)	15(37.5%)	0.000
		1	7(17.5%)	11(27.5%)	18(45%)				1	6(15%)	16(40%)	22(55%)	
		2	0	8(20%)	8(20%)				2	0(0%)	3(7.5%)	3(7.5%)	
	Posterior Segment	0	8(20%)	0(0%)	8(20%)	0.000		Posterior segment	0	12(30%)	0	12(30%)	0.000
		1	12(30%)	6(15%)	18(45%)				1	8(20%)	8(20%)	16(40%)	
		2	0	14(35%)	14(35%)				2	0	12(30%)	12(30%)	

MIDDLE LOBE	Medial segment	0	12(30%)	1(2.5%)	13(32.5%)	0.000		Superior lingular segment	0	14(35%)	3(7.5%)	17(42.5%)	0.01
		1	8(20%)	10(25%)	18(45%)				1	6(15%)	10(25%)	16(40%)	
		2	0	9(22.5%)	9(22.5%)				2	0	7(17.5%)	7(17.5%)	
	Lateral segment	0	12(30%)	2(5%)	14(35%)	0.000		Inferior lingular segment	0	11(27.5%)	2(5%)	13(32.5%)	0.000
		1	7(17.5%)	6(15%)	13(32.5%)				1	9(22.5%)	8(20%)	17(42.5%)	
		2	1(2.5%)	12(30%)	13(32.5%)				2	0	10(25%)	10(25%)	

LOBE LOWER	segment	0	8(20%)	0	8(20%)	0.000	LOBE LOWER	Superior segment	0	11(27.5%)	0	11(27.5%)	0..000
		1	11(27.5%)	8(20%)	19(47.5%)				1	8(20%)	10(25%)	18(45%)	
		2	1(2.5%)	12(30%)	13(32.5%)				2	1(2.5%)	10(25%)	11(27.5%)	
	Anterior segment	0	11(27.5%)	1(2.5%)	12(30%)	0.000		Anterior segment	0	11(27.5%)	1(2.5%)	12(30%)	0.000
		1	9(22.5%)	9(22.5%)	18(45%)				1	9(22.5%)	7(17.5%)	16(40%)	
		2	0	10(25%)	10(25%)				2	0	12(30%)	12(30%)	
	Lateral segment	0	9(22.5%)	0	9(22.5%)	0.000		Lateral segment	0	6(15%)	0	6(15%)	0.01
		1	10(25%)	6(15%)	16(40%)				1	11(27.5%)	6(15%)	17(42.5%)	
		2	1(2.5%)	14(35%)	15(37.5%)				2	3(7.5%)	14(35%)	17(42.5%)	
	Medial Segment	0	7(17.5%)	0	7(17.5%)	0.02		Medial segment	0	11(27.5%)	1(2.5%)	12(30%)	0.000
		1	11(27.5%)	10(25%)	21(52.5%)				1	8(20%)	7(17.5%)	15(37.5%)	
		2	2(5%)	10(25%)	12(30%)				2	1(2.5%)	12(30%)	13(32.5%)	
	Posterior Segment	0	7(17.5%)	0	7(17.5%)	0.000		Posterior segment	0	7(17.5%)	0	7(17.5%)	0.000
		1	11(27.5%)	6(15%)	17(42.5%)				1	10(25%)	5(12.5%)	15(37.5%)	
		2	2(5%)	14(35%)	16(40%)				2	3(7.5%)	15(37.5%)	18(45%)	

**Graph-1:**
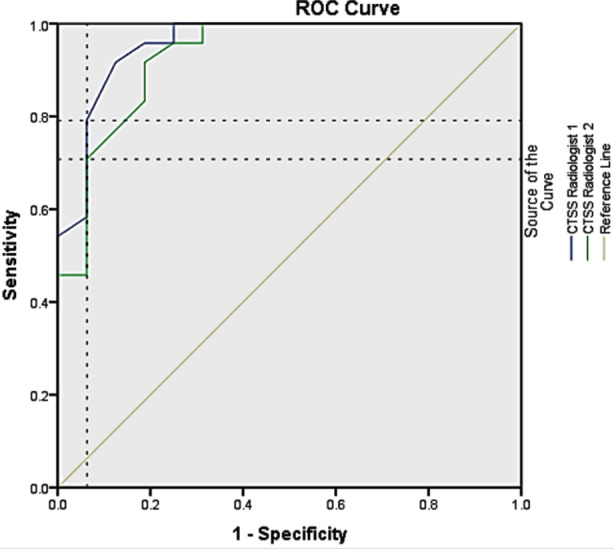
ROC curve indicating the threshold value for two radiologists.

The ROC curve analysis showed threshold values of CT-SS for identifying severe disease as 18.50 and 20.50 with sensitivity of 79.2% and 70.8%, while specificity was 93.7% for both observers. The area under the curve was 0.957 and 0.931 for first and second radiologists respectively (Graph-1). An inter- rater reliability score of 0.994 shows consistency among observers.

## DISCUSSION

As the COVID-19 is still prevailing worldwide with emergence of second, third and now fourth wave in most of the countries, it is mandatory to procure the reliable diagnostic services. Availability and timely utilization of these tools is essential for clinical experts so that early diagnosis and treatment strategies can be planned accordingly. HRCT is useful in understanding extent of damage to lungs due to coronavirus with sensitivity of 98% in contrast to RT-PCR sensitivity of 71%.^16^

Present study showed mixed GGO and consolidation in 19(47.5%), GGO alone in 15(37.5%) and vascular enlargement in 15(37.5%) in sub-pleural posterior distribution (92.5%) as the predominant feature. The reason for GGO alone for being second prevalent feature is that majority of patients had CT done in late stage of disease when GGO alone is less frequently seen. Due to lack of awareness about the early utility of CT scan, very few patients were being imaged in less than two days’ time. Song et al. described pure GGO in 77%, GGO with consolidation in 59% and pure consolidation in 51% with bilateral lung involvement (86%) in sub-pleural posterior distribution mainly in lower lungs.^17^ Zhoa et al. reported GGO alone, combined GGO and consolidation and vascular enlargement in 87(86.1%), 65 (64.4%) and 72(71.3%) patients respectively.^18^

**Fig.2:**
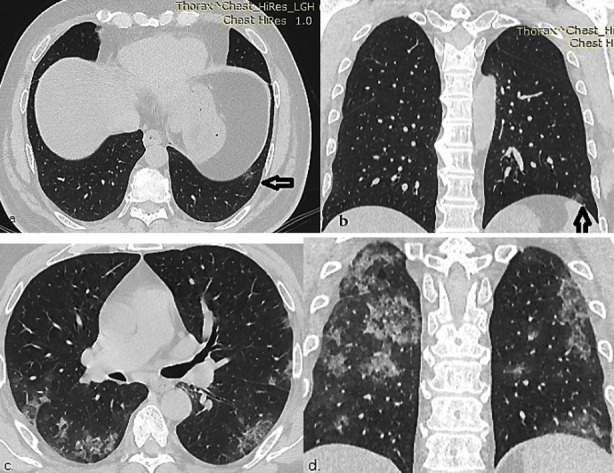
**a- b)** HRCT chest, axial and coronal images at 2^nd^ day of symptom shows unilateral focal GGO in left lateral basal segment. **c-d)** Axial and coronal of same patients on follow-up after 6 days shows bilateral GGO with surrounding rim of consolidation giving reverse halo sign in peripheral posterior distribution.

HRCT findings were initially GGO alone (50%) which changed into combined GGO and consolidation (44.4%) in progressive stage in the current study Fig.2. The unilateral findings in sub-pleural distribution at onset of symptoms changed into bilateral changes involving peri-hilar (44.8%), bronchovascular (69%) and diffuse (20.7%) distribution in late stage.^19^ Zhou et al. described fever in 87%, cough in 56% and dyspnea in 27% cases. He also reported GGO and reticular pattern (58%) along with GGO and consolidation in early progressive stage (1-7 days), while increase frequency of sub-pleural lines, fibrotic stripes, GGO and consolidation in advanced stage (8-14 days).^20^

Bernheim et al. reported linear opacity, crazy paving and reverse halo in 20%, 20%, and 4% in late stage. Total 56% patients in early stage had normal CT without any GGO or consolidation. The most frequent lobe involvement was lower lobe(Right 65%, Left 63%)and left upper lobe in 48%.^12^,^21^ Current study showed high frequency of sub segmental consolidation(51.7%) following air bronchogram consolidation (34.5%) in late stage .Zhou *et al* in another study in 62 patients in Wuhan ,China reported a significant difference in early and late phase CT findings of ground glass opacity (47% vs 27%),ground glass with reticular opacity (50% vs 86%) and air bronchogram (62% vs 90% respectively).^22^

Yang et al. on analysis of ROC curve reported 19.5 as threshold value for severe disease with sensitivity of 83.3% and specificity of 94% (18.5 and 20.5 threshold value for two radiologists in current study). Posterior basal segment of lower lobe (right 81.4%, left 79.4%), left lateral basal segment (77.5%) were frequently involved segment. There was higher scoring in severe group than in mild group. Both these studies also shared more involvement of lower lobes than upper lobes in each group.^15^ These scores in correlation with clinical symptoms helps in predicting clinical outcome and mortality rate at an early stage.^23^

### Limitations of the study:

It includes small sample size and non-availability of serial CT scans in majority of patients. Only few patients were imaged in early stage due to concept of utilization of CT in only detecting complications of COVID-19 at late stage. Asymptomatic patients which pose a large infectivity burden were also not included.

## CONCLUSION

HRCT chest evolving characteristics are effective in understanding variation in pattern of coronavirus disease. Identification of imaging patterns with respect to infection time course is an effective paramount for disease diagnosis, understanding progression and potential complications of disease. CT-SS plays an important role in predicting disease severity, prognosis and clinical outcome. We recommend that early HRCT chest scan will leads to better diagnosis and management of COVID-19 patients.

### Author Contributions:

**NI:** Conceived the idea and study design, data acquisition, analysis, interpretation, content writing, final approval of version, accountable for accuracy of work.

**NH:** Data acquisition, interpretation, content writing and revised the article critically.
